# MiR‐548 K regulatory effect on the *ABCG2* gene expression in MDR breast cancer cells

**DOI:** 10.1002/cnr2.1816

**Published:** 2023-05-11

**Authors:** Mohammadreza Saberiyan, Zahra Ghasemi, Hajar Yaghoobi

**Affiliations:** ^1^ Cellular and Molecular Research Center, Basic Health Sciences Institute Shahrekord University of Medical Sciences Shahrekord Iran; ^2^ Department of Medical Biotechnology, School of Advanced Technologies Shahrekord University of Medical Sciences Shahrekord Iran; ^3^ Clinical Biochemistry Research Center, Basic Health Sciences Institute Shahrekord University of Medical Sciences Shahrekord Iran

**Keywords:** *ABCG2*, breast cancer, miR‐548 k, multi drug resistance

## Abstract

**Background:**

multidrug resistance (MDR) is One of the foremost challenges in overcoming breast cancer. Various molecular processes are involved in the development of MDR in breast cancer cells, including over expression of ABC transporters such as *ABCG2* (*BCRP*), increase breast cancer stem cells drug resistance, and epithelial mesenchymal transition.

**Aims:**

In the present study, we used bioinformatics and experimental analysis to investigate the role of miR‐548 K, in the modulating of *ABCG2,* in MDR breast cancer cells.

**Methods and Results:**

In silico inspections introduce 14 microRNAs targeting 3′‐UTR region of ABCG2 transcripts, which are probably involved in breast cancer drug resistance. An association was highlighted between miR‐548 k with ABC transporter family. The expression level of *ABCG2* gene in MCF7‐MX cell lines was significantly more than MCF7 cell lines. On the other hand, we increased the expression of miR‐548 K in MCF7‐MX and MCF7 cell lines through its transfection, which dramatically coincided with decreasion in the *ABCG2* transcripts level. Additional studies on patient samples revealed that the expression of *ABCG2* showed an increase in *ABCG2* level in neoadjuvant chemotherapy drugs resistance (NCDR) patients compared to primary pre‐operative chemotherapy drugs response (PCDR) patients. Also, a reduction in the expression of miR‐548 K in NCDR patients was revealed.

**Conclusion:**

The results of our study suggest that miR‐548 K may be involved in modulating the expression of *ABCG2* in MDR breast cancer cells.

## 1 INTRODUCTION

Breast cancer is known as the most common cancer among women. This condition is a multifactorial disease which various factors involved in its development.^1^ Genetic, epigenetic, environmental, and lifestyle major factors involved in cancer development. Genomic mutations, chromosomal instabilities, genomic imprinting, and non‐coding RNAs are the most significant genetic and epigenetic factors in tumorigenesis.^2^, ^3^

Cancer cells acquire various properties that cause cell growth and proliferation as well as resistance to stresses in later stages. Some principal problems in cancer treatment are the transformation of cancer cells and adaptation to the conditions and stresses imposed on them. Multi‐drug resistance (MDR) is a challenge in overcoming cancer cells. In breast cancer, chemotherapy is a treatment strategy employed before surgery to limit the tumor cells (neoadjuvant therapy) or after surgery to prevent the recurring growth of cancer cells (adjuvant therapy). Drug resistance may be observed at any of these steps. Various mechanisms are involved in the development of drug resistance; the most well‐known is the overexpression of drug transporters like the ATP‐binding cassette (ABC), which are located on the cytoplasmic membrane.^4^ ABC transporters have similar transmembrane domains that can pump out the chemotherapy drugs in an energy‐dependent ATP manner from cancer cells. ATP‐binding cassette, subfamily G, member 2 (ABCG2), also known as breast cancer resistance protein (BCRP), is a member of the drug transporter family whose role in MDR ​​is known.^5^ It was shown that in the later stages of breast cancer, which is associated with drug resistance, *ABCG2* gene expression increases, which leads to a more frequent appearance of this protein on the cell membrane. This rise in the level of ABCG2 protein helps pump out the chemotherapy drugs from the cancer cells thereby causing drug resistance. Although ABCG2 role in the development of drug resistance in cancer cells is known, the regulatory mechanisms that enhance the expression of this protein in cancer cells and create a growth advantage have not yet been identified.^6^, ^7^, ^8^, ^9^, ^10^

MicroRNAs are small non‐coding RNAs that have been shown to play a role in various cellular and pathogenic processes. Changes in the miRNAs expression patterns in different diseases, including cancer, are tracable; this feature introduces the miRNAs as a potential biomarker.^11^, ^12^ Recent studies have shown the role of microRNAs in cancer drug resistance (CDR). So far, many miRNAs have been reported to be involved in regulating the expression of the ABC transporter family genes.^13^, ^14^ MiR‐548 k is one of the most effective miRNAs in regulating the expression of genes involved in breast cancer and has been suggested to be possibly involved in ABC transporters ‐ dependent MDR.^4^

In the present study, we aimed to investigate the role of miR‐548 k on *ABCG2* gene expression in breast cancer cells.

## 2 MATERIALS AND METHODS

**MicroRNA selection** MicroRNAs that have a protected target site on *ABCG2* mRNA were identified using the miRTarBase (https://mirtarbase.cuhk.edu.cn/), TargescanHuman8.0 database (www.targetscan.org) and, miRWalk (http://mirwalk.umm.uni-heidelberg.de/). MicroRNAs tissue expressions were checked by the GTEx database (www.gtexportal.org). Pubmed (https://pubmed.ncbi.nlm.nih.gov), Scopus (https://www.scopus.com), COREMINE (https://www.coremine.com), and google scholar (http://scholar.google.com) were used to scrutinize the published studies. Basic Local Alignment Search Tool (BLAST) (https://blast.ncbi.nlm.nih.gov/) was used to align the sequences. A network designed by performed by Cytoscape v3.9.0.

### 2.1 Cell lines

Human breast cancer cells MCF‐7 and the multidrug‐resistant cell line MCF7‐MX obtained from American Type Culture Collection (ATCC, Manassas, VA, USA), were provided from the cell bank at the Pasteur Institute (Tehran, Iran). These cells were cultured in Dulbecco's Modified Eagle Medium (GIBCO, Grand Island, NY) containing 10% fetal bovine serum (GIBCO), 1% Antibiotic‐Antimycotic (100×) (Gibco), and MX only to the culture media for MCF‐7/MX cells in an atmosphere with 5% CO_2_ at 37°C.

### 2.2 Cell transient transfection

MCF‐7 and MCF‐7/MX cells were seeded into 6‐well plates (3 × 10^5^/well), incubated for 24 h, and then transfected with 25 nM miR‐548 K mimic, 25 nM miR‐NC using iN‐fect™ (iNtRON biotechnology, Korea), according to the manufacturer's instructions. Briefly, the reagent and FBS were diluted in a 1:30 ratio then adding the target (miR‐548 K or scramble oligonucleotides) was incubated for 15 min at room temperature. Finally, the iN‐fect/oligonucleotide complex was poured gently on the cells and returned to the incubator for 24–48 h. The transfection efficiency was set up by scramble oligonucleotide labeled with FITC, and results were observed using a fluorescent microscope (Nikon, AZ100, Japan). Forty‐eight hours after transfection, the cells were used for RNA extraction.

### 2.3 Patients and samples

Nineteen fresh breast cancer tissue samples were collected from Seyed Shohada Isfahan Hospital and Kashani Hospital (Shahrekord, Iran) including 10 patients with stage III breast tumors and pre‐operative neoadjuvant chemotherapy drugs resistance (NCDR) (including mitoxantrone/doxorubicin/cyclophosphamide) along with 9 patients with stage I breast tumors and primary pre‐operative chemotherapy drugs response (PCDR). Normal breast tissue was obtained from areas adjacent to the tumor tissue as control. Patients who did not receive pre‐operative chemotherapy or suffered from other malignancies were excluded from this study. According to The protocols of the Ethics committee of SKUMS (IR.SKUMS.REC.1399.197 and IR.SKUMS.REC.1399.142), after achiving informed consent, clinicopathological data were received from all the patients. The study protocol also complied with the Declaration of Helsinki (Association, 2019). Tissue biopsies were snap‐frozen and stored at −80°C until RNA extraction.

### 2.4 Quantitative real‐time PCR (QRT‐PCR)

Quantitative Real‐Time PCR was performed to detect the expression levels of the *ABCG2* and miR‐548 K. Total RNA was extracted from cultured MCF‐7 and MCF‐7/MX cells and patient samples with triazole (Sina colon, Iran) method. Total RNA quality and concentration were evaluated by electrophoresis in a 1.5% agarose gel, and D260/D280 and D260/D230 absorbance by Thermo Scientific™ NanoDrop 2000. DNase I (Cat.no: EN0521; Thermo Fisher Scientific, USA) was used to remove DNA.

*ABCG2* cDNA synthesis was performed using Oligo dT primers, according to the manufacturer's protocol for the Geneall Kit. QRT‐PCR was performed by Rotor‐Gene Q (Corbett Research, Sydney, AU) using 2X QPCR Master Mix Syber Green (yektatajhiz, Iran). The GAPDH was used as an internal control for data normalization. The following PCR amplification primers were used for *ABCG2* and *GAPDH*:

*ABCG2*: Forward, 5′‐ GTTTCAGCCGTGGAAC‐3′; and Reverse, 5′‐ CTGCCTTTGGCTTCAAT‐3′.

*GAPDH*: Forward, 5′‐ TTCACCACCATGGAGAAGGC‐3′; and Reverse, 5′‐CCCTTTTGGCTCCACCCT‐3′.

For miR‐548 K cDNA synthesis, miRNAs polyadenylation was done and subjected to reverse transcription using BON‐miR 1st‐strand cDNA synthesis kit and then QRT‐PCR was performed using BON‐miR QPCR (Stem Cell Technology, Iran), according to the manufacturer's protocol. Small Nucleolar RNA, C/D Box 47 (SNORD47) was used as an internal control for data normalization. The Pfaffl method was used to report fold changes.^15^

### 2.5 Statistical analysis

The REST 2009 software (Qiagen, Hilden, Germany) was used for relative expression analysis.The quantities of mRNA and miRNA in the tissues or cell lines were standardized to the *GAPDH* mRNA and *SNORD47* respectivly. Comparation between tumor or treatment mimic and adjacent normal breast tissues or treatment scrambles cell lines were done respectivly. Futhermore, for explain the association between genes or miRNA expressions in a certain sample, the results were stated as the ratio of target genes over *GAPDH* or *SNORD47* expression (relative expression [REx]) respectivly. QRT‐PCR were performed in triplicate for each sample, and the results were averaged. The statistical analyses of the expression data were performed by Graph Pad prism 8.0. The t‐test was used to obtain the rate of change of expression data. *P*‐value < .05 was considered significant.

## 3 RESULTS

### 3.1 miR‐548 k as a regulatory factor for the ABC transporter family

**FIGURE 1:**
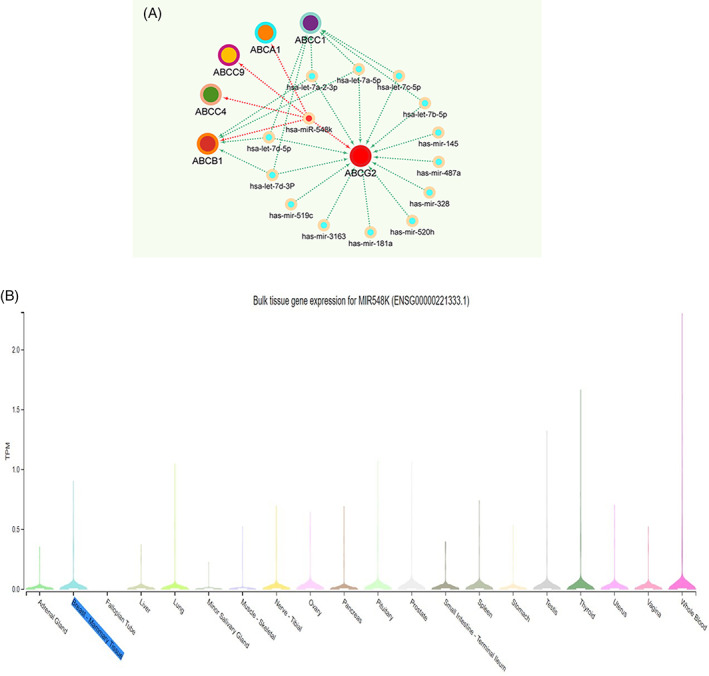
MicroRNAs and breast cancer. MicroRNAs that are probably involved in breast cancer drugs resistance mediated by ABCG2 and miR‐548 k connection with other members of the ABC transporter family. The Cytoscape software was used to make the visualization of the network (A). The has‐mi‐R548k expression in breast tissue according to the GTEX database (B).

Extracted data related to *ABCG2*‐related microRNAs from miRTarBase, TargescanHuman8.0 and miRWalk databases showed that 1100 microRNAs have target sites on the 3′‐UTR region of the transcripts of this gene. Tissue expressions were checked by the GTEx database (www.gtexportal.org); the microRNAs that did not have expression in the breast tissue were excluded from the study. In the next step, we checked the filtered microRNAs in terms of their expression in breast tissue, in the published documents, and in terms of their role in drug resistance in breast cancer. Fourteen microRNAs were determined, which may probably be involved in breast cancer drug resistance mediated by ABCG2 (Fig 1A). One of these microRNAs is miR‐548 k which has three targets site in positions 494–500, 2736–2742, and 5197–5203 of *ABCG2* 3’‐UTR region (Figure 1B) (Table 1). An association was deciphered between miR‐548 k and some other members of ABC transporter family that involved in drug resistance (Figure 1A).

**TABLE 1:** MiR‐548 k target sites in *ABCG2* 3’ UTR

	Position	Predicted consequential pairing of target region (top) and miRNA (bottom)
1	Position 494–500 of *ABCG2* 3’ UTR	5′ …GCCCUUCAGUCUUAAUACUUUAU… |||||| 3’ UCGUUUUAGGCGUUCAUGAAAA
2	Position 2736–2742 of *ABCG2* 3’ UTR	5′ …CCUUAAGUAAAUUUCUACUUUAU… |||||| 3’ UCGUUUUAGGCGUUCAUGAAAA
3	Position 5197–5203 of *ABCG2* 3’ UTR	5′ …GCCUGUUUGUAUACAUACUUUAA… |||||| 3’ UCGUUUUAGGCGUUCAUGAAAA

### 3.2 Increased miR ‐548 k expression is associated with decreased *ABCG2* gene expression

**FIGURE 2:**
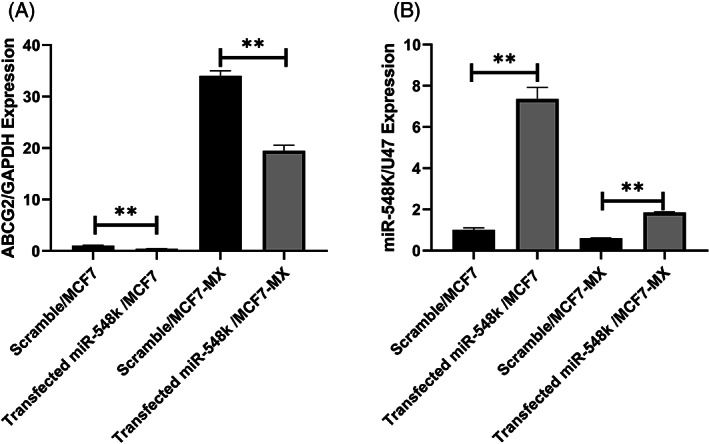
MiR‐548 k and ABCG2 expressions in in transfected miR‐548 k/MCF7 and miR‐548 k /MCF7‐MX. ABCG2 expression in transfected miR‐548 k/MCF7 (*P* = 0.003; ** pronouncedly significant) and miR‐548 k /MCF7‐MX (*P* = 0.001; ** pronouncedly significant) was reduced in comparison with scramble/MCF7 and scramble/MCF7‐MX (A). miR‐548 k expression was increased in transfected miR‐548 k/MCF7 (*P* = 0.002; ** pronouncedly significant) and miR‐548 k /MCF7‐MX (*P* = 0.003; ** pronouncedly significant) in comparison with scramble/MCF7 and scramble/MCF7‐MX (B).

The statistical analyses revealed that the expression of *ABCG2* gene in MCF7‐MX was 34.876 (*P* < .0001) fold greater than in MCF7 cell lines. Also, it was highlighted that the expressions of *ABCG2* gene in treatment mimic MCF7 cell lines was 0.407 (*P* = 0.003) fold and in MCF7‐MX was 0.573 (*P* = 0.001) fold fewer in comparison with treatment scrambles respectively (Fig 2A).

On the other hand, a miR‐548 k expression in treatment mimic MCF7 cell line was 7.359 fold (*P* = 0.002) and in MCF7‐MX cell lines was 3.046 (*P* = 0.003) times more than treatment scrambles respectively (Fig 2B).

### 3.3 Expression of *ABCG2* gene and miR‐548 k in patients with chemotherapy drug resistance

**FIGURE 3:**
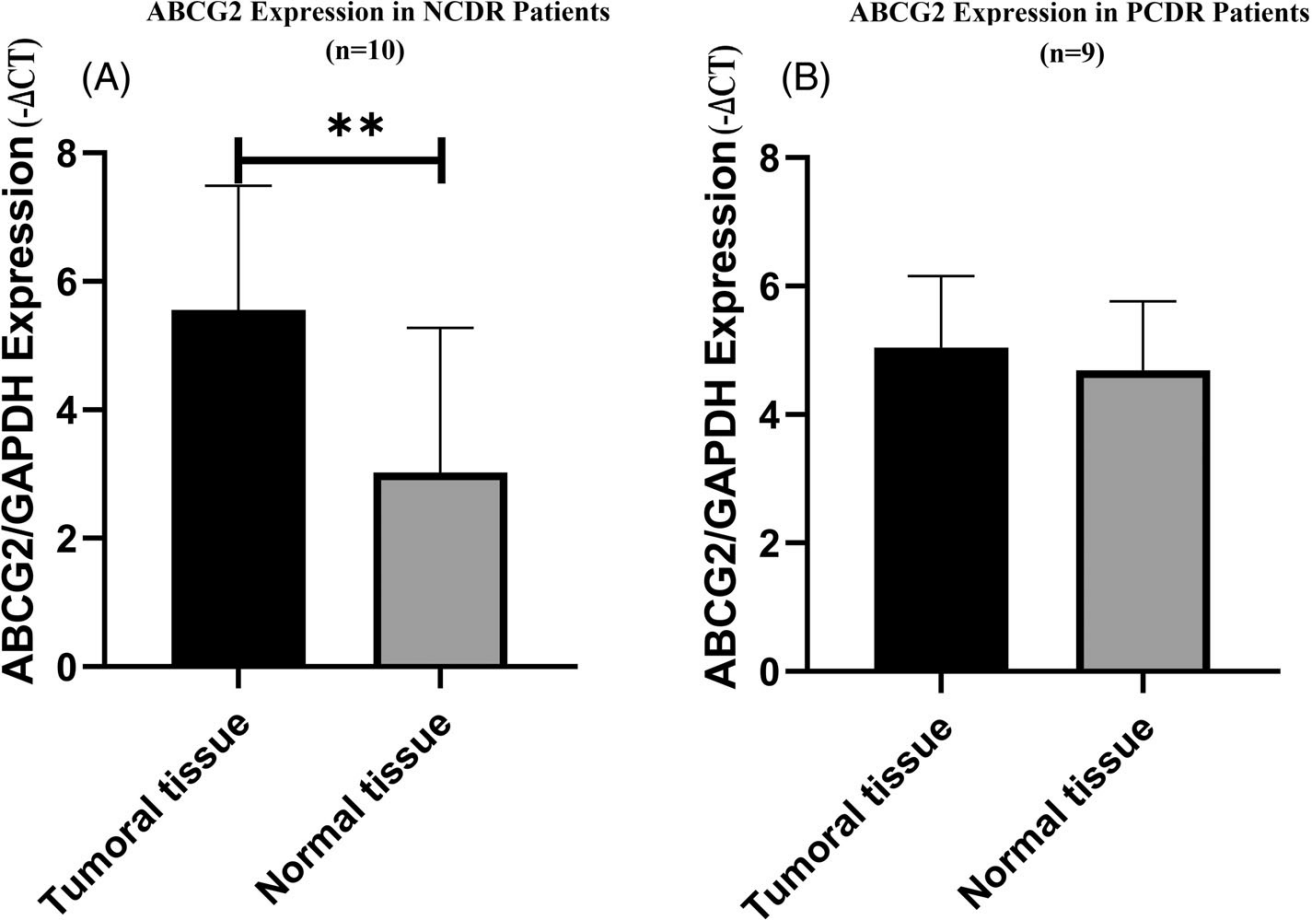
ABCG2 gene expression in NCDR and PCDR patients. ABCG2 gene expression was more (*P* = 0.002; ** pronouncedly significant) in tumor tissue in comparison with adjacent normal tissue in NCDR patients (*n* = 10)(A). The changes of ABCG2 gene expression in tumor tissue was not significant than adjacent normal tissue in PCDR patients (*n* = 9)(B).

**FIGURE 4:**
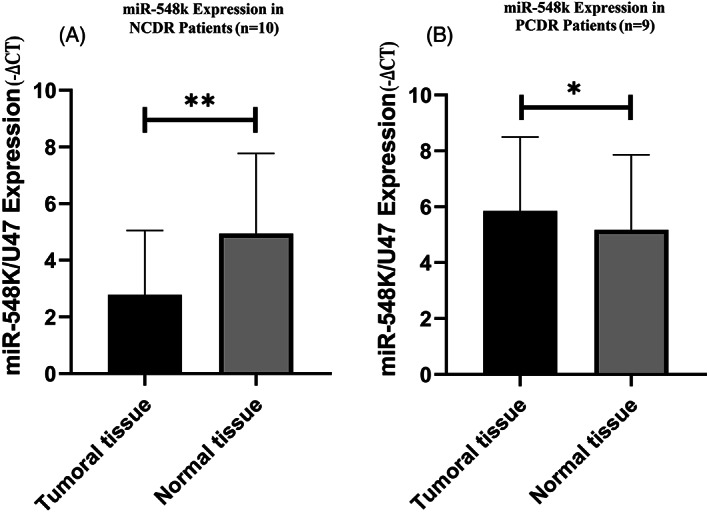
MiR‐548 k expression in NCDR and PCDR patients. miR‐548 k expression was lower (*P* = 0.002; ** pronouncedly significant) in tumor tissue in comparison with adjacent normal tissue in NCDR patients (*n* = 10)(A). miR‐548 k expression in tumor tissue was more (*P* = 0.019; * significant) than adjacent normal tissue in PCDR patients (*n* = 9)(B).

Expression of *ABCG2* gene in tumor tissues in NCDR patients was 7.582 fold (*P* = 0.002) more than in the adjacent normal tissues but the expression changes of *ABCG2* gene in tumor tissues in PCDR patients were not significant in comparison with adjacent normal tissues (Figure 3A, B).The results indicated that the expression of miR‐548 k in tumor tissues in NCDR patients was 2.840 times (*P* = 0.002) lower in comparison with adjacent normal tissues, but the expression of miR‐548 k in tumor tissues in PCDR patients was 1.727 times (*P* = 0.019) more in comparison with adjacent normal tissues (Figure 4A, B).

## 4 DISCUSSION

It is renowned that the ABCs transporter family is one of the most prominent transporters, which export toxic substances from inside the cells. Understanding the factors that regulate the expression of these proteins can provide a perspective for overcoming drug resistance. In this study, the results of 4 databases miRTarBase, TargescanHuman8.0, miRWalk, and GTEx identified 14 potential microRNAs as regulators of *ABCG2* gene expression. Meanwhile, miR‐548 k was predicted as one of the most significant microRNAs in the interaction and regulation of gene expression of the ABC transporter family, including ABCB1, ABCC1, and ABCG2, which are involved in MDR. The results of measuring the expression of *ABCG2* gene in MCF7‐MX cell lines were significantly greater than in MCF7 cell lines. On the other hand, the expression of miR‐548 K increased in MCF7‐MX and MCF7 cell lines through its transfection, which dramatically coincided with decreasing in the *ABCG2* gene expression level. Furthermore, studies on patient samples showed that the expression of *ABCG2* elevated in NCDR patients, compared with PCDR patients. Also, a decline in the expression of miR‐548 K in NCDR patients was observed.

Previous studies confirmed the elevation in *ABCG2* gene and protein expression levels in patients with neoadjuvant chemotherapy and invasive breast cancer. Diana E Baxter et al. studies revealed that *ABCG2* was upregulated in breast cancers after treatment with neoadjuvant endocrine therapy that resulted in adjuvant chemotherapy drug resistance.^16^ Also, Yan Shou Zhang et al. revealed that high ABCG2 protein expression is associated with invasive breast cancer, lymphatic metastasis, tumor size, and poor pCR.^17^ TenghuaYu et al. showed that the expression of *ABCG2* is significantly upregulated in tamoxifen‐resistant ER + metastases compared to primary breast tumors.^18^

The function of miR‐548 in regulating different genes was reported. For example, the secretion of VEGF‐C increase with the overexpression of miR‐548 k. Also, miR‐548 k stimulates lymph angiogenesis by regulating ADAMTS1/VEGF‐C/VEGFR‐3 pathways. ADAMTS1 hosts a complementary 3’‐UTR site for miR‐584 k seed region, which upon binding, resulted in down‐regulation of ADAMTS1. However, overexpression of miR‐548 k lead to an increase in releasing VEGF‐C from the ADAMTS1 complex, leading to higher initiation of lymph angiogenesis in esophageal squamous carcinoma.^19^ Zhiyao Chen et al. reported that miR‐548 k upregulation promotes the progression of esophageal cancer by targeting lnc RNA‐LET.^20^

On the other hand, the role of miR‐548 in inhibiting the metastasis, proliferation, and migration of breast cancer cells was identified. Yafei Shi et al. studies revealed that miR‐548 functions as an anti‐oncogenic regulator in breast cancer by regulating the expression of ECHS1 and in following inhibiting the proliferation of breast cancer cells.^21^ Also, Pei‐Yi Tan et al. researches demonstrated that miR‐548 could inhibit the proliferation, migration, invasion, and overall progression of breast cancer cells by targeting E2F2.^22^

Prior to this, studies have shown the role of miRNAs in regulating the expression of ABC transporter family genes, which are usually increased when drug resistance develops in cancer cells. A study by BC Gomes et al. showed that the expression of miR‐548 K in doxorubicin‐resistant cancer cells increased after 16 weeks of doxorubicin resistance withdrawal; however, the expression of ABCB1 protein diminished. It was suggested that ABC transporters' family is a target for microRNA‐ mediated gene regulation, thus playing a role in the kinetics of MDR.^4^ On the other hand, YZ pan et al. studies revealed that the expression of miR‐328 negatively regulated the expression of *ABCG2* in human breast cancer cells.^23^ Also, it was suggested that miR‐519c would affect *ABCG2* expression in MCF‐7 human breast cancer cells, and the presence of miR‐519c target site on *ABCG2* 3′UTR may control the *ABCG2* mRNA expression by getting involved in mRNA cleavage mechanism.^24^ The miR‐548 k appears to be a prime factor in tumor signaling pathways. It seems miR‐548 k has a dual role in the development of cancer cells; primarily, increasing the expression of this miRNA can promote the development and growth of cancer cells,^20^ and on the other hand, reducing its expression can lead to the occurrence of drug resistance.^4^ Considering the results of enhanced expression of miR‐548 K due to its transfection in drug‐resistant cancer cells, which led to a decline in the expression of *ABCG2* gene in these cells, as well as the results of miR‐548 K and ABCG2 transcript alignment revealing 3 binding site for miR‐548 K on *ABCG2* mRNA 3′UTR, the role of miR‐548 k in regulating *ABCG2* gene expression was highlighted. One of the probable functions of miR‐548 k is binding to the *ABCG2* gene transcript and directing it to the cleavage pathway, similar to what happens with miR‐519c. But this possibility requires investigation on more samples by more specific techniques to investigate the possible interaction of miR‐548 k, but unfortunately, in this study, we were limited in terms of conducting complementary studies and more functional assays. Overall, miR‐548 k may has the potential to be investigated as a therapeutic and pharmaceutical target for ABCG2‐related MDRs, and future studies with a large study population and functional assay technics will be beneficial.

## 5 CONCLUSION

The results of our study suggested that miR‐548 K may be involved in regulating the expression of *ABCG2* in breast cancer cells. MiR‐548 k likely acts as an inhibitory factor of *ABCG2* transcript in breast cancer cells. Future studies in a larger statistical population with the functional assay can provide a clear perspective to raise it as a therapeutic and pharmaceutical target for overcoming MDR in breast cancer.

## AUTHOR CONTRIBUTIONS

**Mohammadreza Saberiyanm:** Conceptualization (equal); formal analysis (equal); writing – review and editing (equal). **Zahra Ghasemi:** Data curation (equal); visualization (equal); writing – review and editing (equal). **Hajar Yaghoobi:** Conceptualization (equal); funding acquisition (equal); writing – review and editing (equal).

## FUNDING INFORMATION

This study was financially supported by the Shahrekord University of Medical Sciences (grant no: 3417 and 3529).

## CONFLICT OF INTEREST STATEMENT

The authors have stated explicitly that there are no conflicts of interest in connection with this article.

## ETHICS APPROVAL AND CONSENT TO PARTICIPATE

According to The protocols of the Ethics committee of SKUMS (IR.SKUMS.REC.1399.197 and IR.SKUMS.REC.1399.142), clinicopathological data were received from all the patients. The study protocol was also complied with Declaration of Helsinki (Association, 2019).

## Data Availability

Data sharing is not applicable to this article as no new data were created or analyzed in this study.
